# Activity of the Highly Specific RET Inhibitor Selpercatinib (LOXO-292) in Pediatric Patients With Tumors Harboring *RET* Gene Alterations

**DOI:** 10.1200/PO.19.00401

**Published:** 2020-04-14

**Authors:** Michael V. Ortiz, Ulrike Gerdemann, Sandya Govinda Raju, Dahlia Henry, Steve Smith, S. Michael Rothenberg, Michael C. Cox, Stéphanie Proust, Julia Glade Bender, A. Lindsay Frazier, Peter Anderson, Alberto S. Pappo

**Affiliations:** ^1^ Memorial Sloan Kettering Cancer Center, New York, NY; ^2^ Dana-Farber Boston Children’s Cancer and Blood Disorders Center, Boston, MA; ^3^ Loxo Oncology, Stamford, CT; ^4^ Loxo Oncology, South San Francisco, CA; ^5^ Department of Pediatric Hemato-Oncology, Children’s University Hospital, Angers, France; ^6^ Cleveland Clinic, Cleveland, OH; ^7^ St Jude Children’s Research Hospital, Memphis, TN

## INTRODUCTION

The *RET* gene can be oncogenically activated by point mutations, in-frame deletions, and chromosomal rearrangements,^1,2^ gain-of-function events that render the RET tyrosine kinase constitutively active.^3-5^ In pediatric and young adult patients, *RET* gene fusions have been reported in 22% to 45% of papillary thyroid carcinomas (PTCs)^6-9^ and less frequently in pediatric and young adult patients with glioma,^10^ lipofibromatosis,^11^ inflammatory myofibroblastic tumor,^12^ and infantile myofibromatosis.^13^ In addition, activating point mutations of *RET* have been reported in 40% to 50% of sporadic medullary thyroid cancers (MTCs).^14,15^ If they are constitutional, such mutations lead to the hereditary autosomal-dominant cancer syndrome called multiple endocrine neoplasia type 2 (MEN2), one characteristic of which is predisposition to MTC.^16,17^ Currently, no highly specific RET-targeted agents are approved for the treatment of patients with *RET*-altered cancers.

Selpercatinib (LOXO-292) is a potent, adenosine triphosphate–competitive, highly selective small molecule RET inhibitor with nanomolar potency against diverse *RET* alterations (including anticipated acquired gatekeeper resistance mutations).^18,19^ Preliminary results for selpercatinib in a phase I/II trial (LIBRETTO-001; ClinicalTrials.gov identifier: NCT03157128) are highly encouraging, showing that it is generally well tolerated and has marked antitumor activity in adolescent and adult patients with *RET*-altered cancers, including those with brain metastases and those with tumors resistant to previous multitargeted kinase inhibitors.^20,21^ We report the clinical activity of selpercatinib in five pediatric patients with tumors harboring *RET* alterations, four of whom were ineligible for the selpercatinib clinical trial open at the time their treatment was started because of their young age (younger than 12 years).

## METHODS

### Patients

Given the lack of other treatment options, access to selpercatinib for the four patients ineligible for an ongoing clinical trial was enabled by single patient protocols that were allowed by country-specific regulatory agencies and approved by institutional review boards. All four patients received selpercatinib (capsule or liquid formulation) orally in continuous 28-day cycles at a starting dose of 90 mg/m^2^ twice per day. This dose was intended to deliver exposure equivalent to the recommended adult phase II dose of 160 mg twice per day. One patient was enrolled to the 80-mg cohort of the ongoing selpercatinib phase I/II trial (ClinicalTrials.gov identifier: NCT03157128) and underwent intrapatient dose escalation to 160 mg per protocol.

### Pharmacokinetic Analysis

Serial blood samples were collected for pharmacokinetic analyses. Plasma concentrations of selpercatinib were determined by liquid chromatography with detection by mass spectrometry. Pharmacokinetic parameters were calculated using Microsoft Excel (Microsoft, Redmond, WA)

## RESULTS

The analysis cutoff date was October 1, 2019. Two patients had thyroid cancers and three had soft-tissue sarcomas (Table 1).

**TABLE 1.**
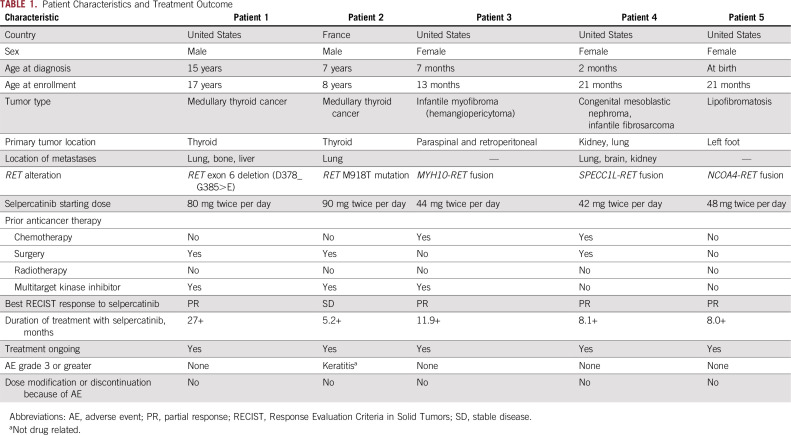
Patient Characteristics and Treatment Outcome

### Thyroid Cancers

#### Patient 1.

**FIG 1.**
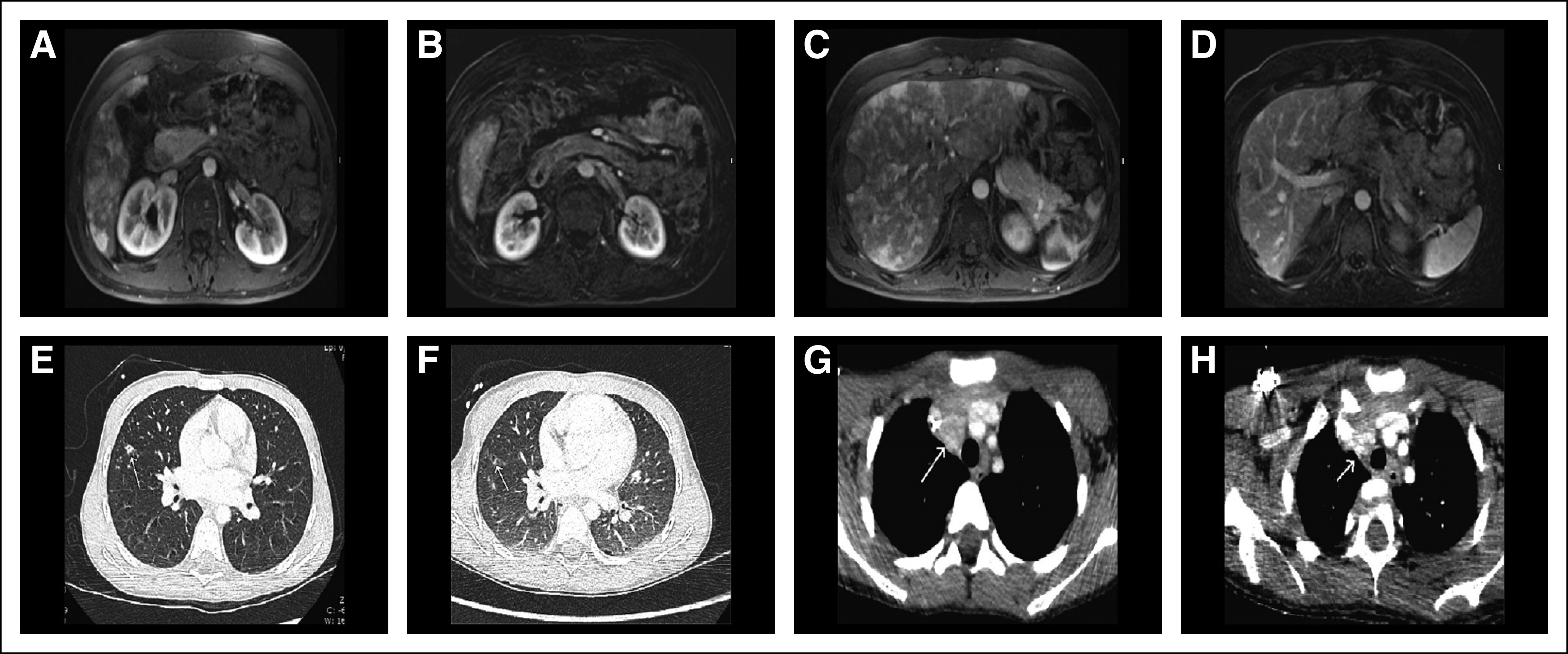
Selpercatinib activity in patients with medullary thyroid cancer. Patient 1: magnetic resonance imaging scans of (A) right lateral and (B) left anterior liver metastatic lesions at baseline and after 22 months of treatment with selpercatinib of (C) right lateral and (D) left anterior in a heavily pretreated patient with RET-mutated medullary thyroid cancer. A rapid improvement in symptoms and a partial response were reported after two cycles of treatment, which deepened over time. Patient 2: computed tomography scans at baseline and after 4 months of treatment with selpercatinib of thorax (E, F, respectively) and mediastinum (G, H, respectively) showing metastatic disease in a patient with *RET*-mutated medullary thyroid cancer. Early disease control was achieved after two cycles of treatment.

A 15-year-old boy presented with symptoms of night sweats, flushing, cramping, and weight loss. A biopsy revealed the presence of an MTC, with metastases detected in the lung, bone, and liver. The patient underwent total thyroidectomy and bilateral neck dissection. Molecular analysis showed that the tumor harbored an *RET* exon 6 deletion (d378_G685>E), and the patient was treated sequentially with four multitargeted kinase inhibitors: vandetanib, sunitinib, cabozantinib, and lenvatinib, which were each discontinued because of either adverse events or lack of efficacy. The patient was subsequently enrolled on the LIBRETTO-001 trial, and selpercatinib was initiated at 80 mg twice per day. After 8 weeks of treatment, a partial response was demonstrated, with a maximum tumor reduction of 86% at 40 weeks (Fig 1A-D). The patient remains in response and in excellent health after 25 cycles of treatment with no adverse events reported. Calcitonin was 53,125 pg/mL at the onset of therapy and is now 555 pg/mL; carcinoembryonic antigen was 1,850 ng/mL at treatment cycle 1 and is now 287 ng/mL.

#### Patient 2.

A 7-year-old boy with neonatal hypotonia, abdominal pain, hollow feet, and unexplained laryngeal spasms was referred for diagnostic exome sequencing of genomic DNA (SureSelect XT Clinical Research Exome, Agilent, Santa Clara, CA). This revealed a constitutional de novo *RET* M918T mutation, a pathogenic variant associated with MEN2B that confers the highest risk of early onset MTC.^16^ The patient was subsequently diagnosed with an MTC and underwent thyroidectomy with tracheostomy followed by vandetanib therapy, which was discontinued because of the onset of grade 3 colitis. Selpercatinib was subsequently initiated at 90 mg twice per day. Treatment-related adverse events included grade 1 vomiting and diarrhea. After two cycles of treatment, stable disease was observed (Fig 1E-H).

### Soft-Tissue Sarcomas

#### Patient 3.

A 7-month-old girl presented with lower extremity paraplegia. Magnetic resonance imaging showed a mass infiltrating the spinal canal, retroperitoneum, and pelvis. Initial pathology revealed clusters of CD56^+^ spindle cells consistent with neuroblastoma. Because of its location, the primary tumor was deemed unresectable.

The patient was started emergently on chemotherapy with cyclophosphamide and topotecan. Treatment was complicated by *Pseudomonas*-associated ecthyma gangrenosum, pneumonia, and bacteremia. Final pathology resulted in a definitive diagnosis of infantile myofibroma/hemangiopericytoma. Magnetic resonance imaging demonstrated new lung nodules indicative of metastatic disease.

**FIG 2.**
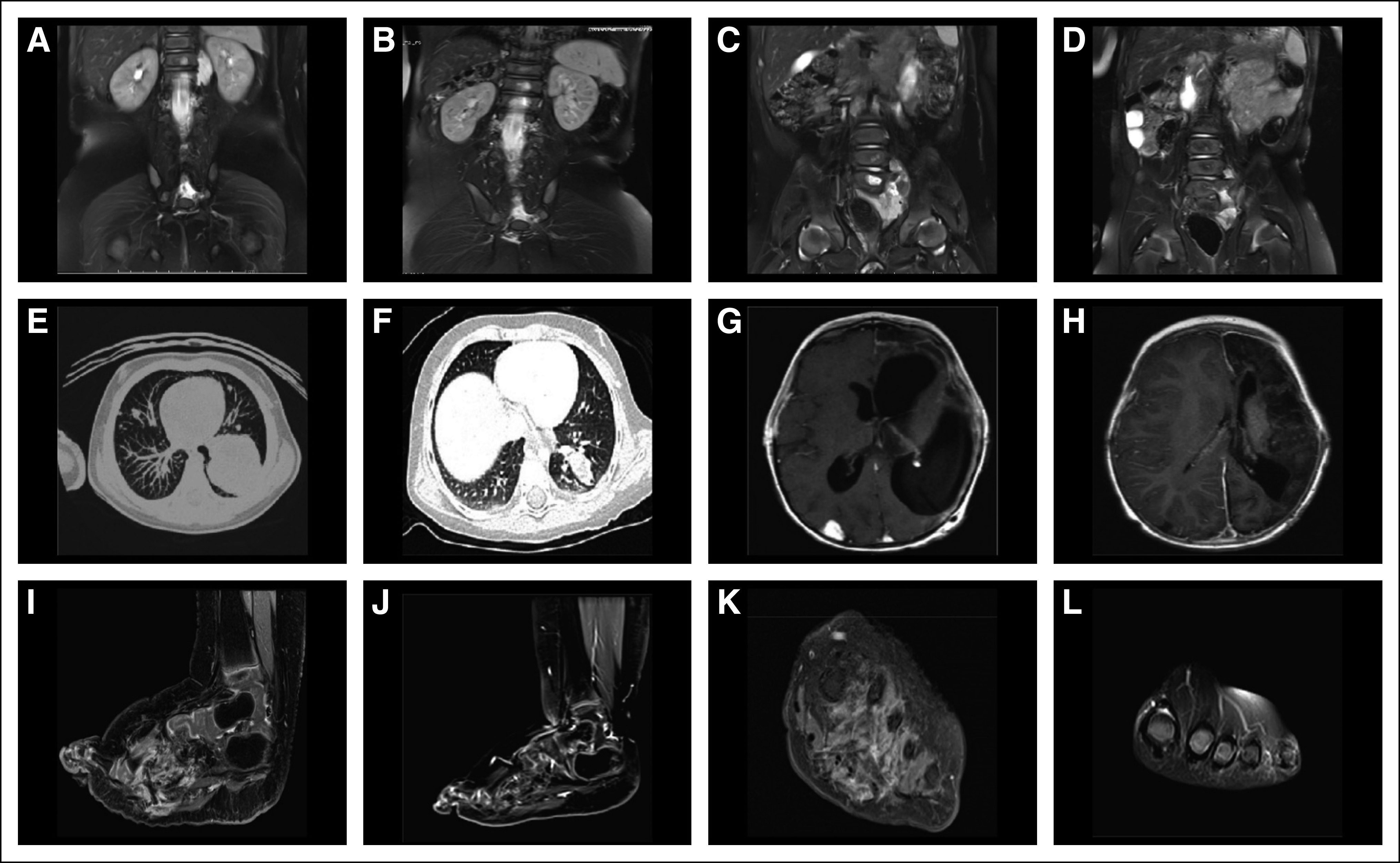
Selpercatinib activity in patients with soft-tissue sarcoma. Patient 3: (A, C) computed tomography (CT) scans of the abdomen at baseline and (B, D) after 6 months of treatment with selpercatinib, revealing multiple paraspinal retroperitoneal and pelvic lesions in a patient with infantile myofibroma/hemangiopericytoma harboring an *MYH10-RET* fusion. A partial response was observed after one cycle of selpercatinib; after six cycles, the paraspinal lesion had completely resolved, and the patient regained lower extremity neurologic function. Patient 4: CT scans at baseline of (E) the lungs and (G) brain and after 8 months of treatment with selpercatinib of (F) the lungs and (H) brain in a patient with an *SPECC1L-RET* fusion–positive congenital mesoblastic nephroma and infantile fibrosarcoma. After two cycles of selpercatinib, a partial response was observed with a 41% tumor reduction, which deepened to 66% by cycle 8. Patient 5: CT scans (I, K) at baseline and (J, L) after 2 months of treatment with selpercatinib of the left foot in a patient with an *NCOA4-RET* fusion–positive lipofibromatosis. Selpercatinib treatment resulted in a significant decrease in tumor burden leading to improvements in gait and locomotion.

An RNA-based next-generation sequencing (NGS) fusion assay (Solid Fusion Assay V2; ArcherDx, Boulder, CO) indicated that the tumor harbored an *MYH10-RET* gene fusion. The patient was started on vandetanib and achieved a partial tumor response by Response Evaluation Criteria in Solid Tumors (RECIST) 1.1.^22^ However, after 4 months of treatment, she developed disease progression at the primary site. Treatment with selpercatinib was subsequently initiated. After 23 days, imaging demonstrated a partial response, which was confirmed 3 weeks later. Repeat imaging after 6 months of treatment showed complete resolution of the paraspinal mass and a 50% decrease in the size of the retroperitoneal, pelvic, and lumbosacral mass (Fig 2A-D). Together with tumor response, the patient regained normal sensation and muscle movement in her lower extremities and was able to stand and walk with support. No adverse events were reported.

#### Patient 4.

During repair of congenital bilateral inguinal hernias, a 2-month-old girl was found to have a right renal mass, and she underwent a right nephrectomy. Pathology identified mesoblastic nephroma (cellular type) with positive surgical margins. The patient initially did well without systemic therapy. She subsequently presented with the sudden onset of inconsolable crying and refusal to bear weight. Abdominal ultrasound and chest x-ray identified a mass in the left kidney and a large left lower lung mass; computed tomography imaging confirmed these lesions and identified additional, multiple small lung lesions. Brain imaging identified a large left-sided cerebral vascular accident and multifocal right posterior brain lesions. Biopsy of a lung mass revealed high-grade spindle cell sarcoma consistent with an infantile fibrosarcoma; pathology review of the original right nephrectomy specimen confirmed the initial diagnosis of mesoblastic nephroma. She received one cycle of actinomycin and vincristine followed by vincristine, dactinomycin, and cyclophosphamide. A subsequent ultrasound scan showed the renal mass to be enlarging. The patient received additional chemotherapy with cyclophosphamide and topotecan, but treatment was complicated by febrile neutropenia and *Enterococcus faecalis* ventriculitis.

DNA-based NGS (using Memorial Sloan Kettering integrated mutation profiling of actionable cancer targets [MSK-IMPACT]) of the tumor from the renal and lung masses identified the same *SPECC1L-RET* gene fusion, and the patient initiated treatment with selpercatinib. After two cycles, a partial response was observed with a 41% tumor reduction, (Fig 2E-H). A selpercatinib concentration of 4.5 ng/mL was achieved in cerebrospinal fluid at a dose level of 48 mg twice per day (90 mg/m^2^ per dose). Upon detection and resection of an isolated metastasis at the right posterior temporal occipital junction, the dose of selpercatinib was increased to 94 mg (180 mg/m^2^) twice per day, raising the concentration of the drug to 16 ng/mL in the cerebrospinal fluid. Responses deepened to 66% by cycle 6, and no treatment-related adverse events were reported.

#### Patient 5.

An otherwise healthy 21-month-old girl had been diagnosed with lipofibromatosis of her left foot at birth. This had progressively increased in size and affected her ability to ambulate. She was evaluated by oncologists and surgeons who recommended amputation. NGS analysis of a biopsy specimen (FoundationOne CDx; Foundation Medicine, Cambridge, MA) identified an *NCOA4-RET* fusion, which was confirmed by whole genome and transcriptome analysis. Selpercatinib was initiated, and imaging after 2 months revealed a partial response by RECIST 1.1, with a 59% reduction in tumor volume and resolution of tumor infiltration of the metatarsals (Fig 2I-L). No adverse events were reported.

##### Pharmacokinetic analysis.

**FIG 3.**
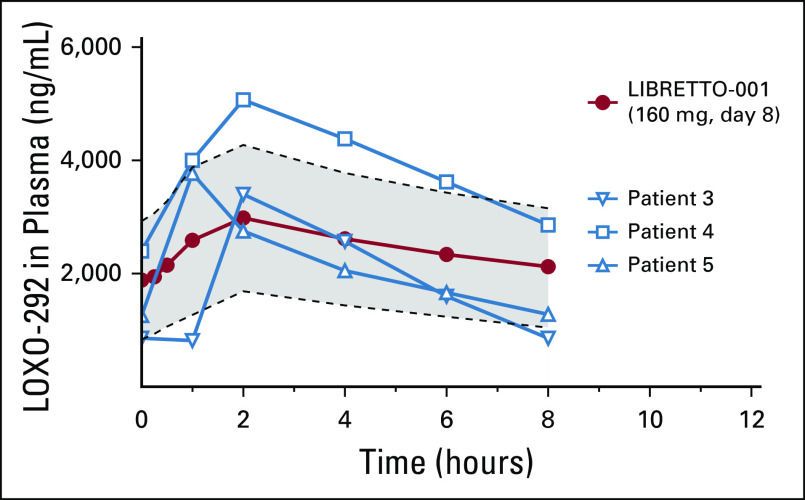
Pharmacokinetics of selpercatinib in children. Plasma samples from three patients revealed that adequate plasma concentrations of selpercatinib were achieved (greater than RET wild-type concentration that inhibits 90% [IC_90_]), which were within the range seen in adult patients treated with the recommended dose of 160 mg in the LIBRETTO-001 trial. The gray area represents the 95% CIs for the median plasma concentrations observed in patients treated in the LIBRETTO-001 trial (red circles).

Selpercatinib pharmacokinetic data are available for three pediatric patients (patients 2, 3, and 4). The estimated steady-state maximum serum concentration (C_max_) and area under the serum concentration-time curve for 24 hours (AUC_0-24_) in these patients was similar to that of adults treated with selpercatinib 160 mg twice per day and consistent with significant (plasma concentrations greater than the concentration that inhibits 90% [IC_90_]) calculated RET target inhibition (Fig 3; data on file, Loxo Oncology, Stamford, CT).^23^

## DISCUSSION

*RET* alterations are actionable oncogenic drivers that occur commonly in MTCs,^24^ pediatric PTCs,^6-9^ and rarely in other pediatric cancers.^10-13,25^ The multitargeted kinase inhibitors cabozantinib, vandetanib, and lenvatinib have demonstrated modest antitumor activity in adult and pediatric patients with MTC,^26-29^ and adult patients with *RET* fusion–positive cancers.^4,30,31^ The clinical activity of these agents is limited by suboptimal RET inhibition and significant toxicity, most likely because of strong inhibition of other kinases such as KDR and VEGFR2.^5,32^

Selpercatinib has demonstrated durable tumor responses and high tolerability in adolescents and adults with *RET*-altered cancers, with response rates of 68% reported in patients with *RET* fusion–positive platinum-pretreated non–small-cell lung cancer,^20^ and 56% in patients with *RET* mutation–positive cabozantinib and/or vandetanib-pretreated MTC.^21^ The results described here in patients with limited treatment options indicate that selpercatinib is also effective and safe in pediatric patients whose tumors harbor *RET* alterations. A phase I/II pediatric trial for patients with advanced *RET*-altered solid or primary CNS tumors is ongoing (LIBRETTO-121; ClinicalTrials.gov identifier: NCT03899792).

## Data Availability

The following represents disclosure information provided by authors of this manuscript. All relationships are considered compensated unless otherwise noted. Relationships are self-held unless noted. I = Immediate Family Member, Inst = My Institution. Relationships may not relate to the subject matter of this manuscript. For more information about ASCO's conflict of interest policy, please refer to www.asco.org/rwc or ascopubs.org/po/author-center. Open Payments is a public database containing information reported by companies about payments made to US-licensed physicians (Open Payments). **Employment:** Loxo Oncology **Employment:** Loxo Oncology **Stock and Other Ownership Interests:** Loxo Oncology, Allergan, Axovant Sciences, Palatin Technologies **Consulting or Advisory Role:** Various, Loxo Oncology **Patents, Royalties, Other Intellectual Property:** Various patents and applications **Travel, Accommodations, Expenses:** Various **Employment:** Loxo Oncology **Stock and Other Ownership Interests:** Loxo Oncology **Employment:** Bayer, Loxo Oncology, Merck KGaA, Amgen, Day One Biopharmaceuticals **Stock and Other Ownership Interests:** Loxo Oncology, Bayer, Merck KGaA, Amgen, Day One Biopharmaceuticals **Patents, Royalties, Other Intellectual Property:** US patent 62/318,041 issued to Loxo Oncology (Inst) **Consulting or Advisory Role:** AbbVie (Inst) **Research Funding:** Celgene (Inst), Merck (Inst), Pfizer (Inst), Amgen (Inst), Ignyta (Inst), Bristol-Myers Squibb (Inst), Eisai (Inst), Novartis (Inst), Eli Lilly (Inst), Loxo Oncology (Inst), Genentech (Inst) **Travel, Accommodations, Expenses:** Novartis, Amgen, Merck, Bayer, Genentech **Uncompensated Relationships:** SpringWorks Therapeutics, Bristol-Myers Squibb **Stock and Other Ownership Interests:** Decibel Therapeutics **Consulting or Advisory Role:** Decibel Therapeutics **Stock and Other Ownership Interests:** Healios **Consulting or Advisory Role:** Enlivity **Patents, Royalties, Other Intellectual Property:** Patent for glutamine and trehalose compositions; priority date 13 September 2013; App 14/470, 545, filed August 27, 2014; patent 61/878,084 issued December 26, 2017 **Other Relationship:** Enlivity **Honoraria:** Bayer, Roche **Consulting or Advisory Role:** Merck, Loxo Oncology/Bayer, EUSA Pharma No other potential conflicts of interest were reported.
